# Dual Transcriptomics To Determine Gamma Interferon-Independent Host Response to Intestinal Cryptosporidium parvum Infection

**DOI:** 10.1128/iai.00638-21

**Published:** 2022-02-17

**Authors:** Gina M. Gallego-Lopez, Carolina Mendoza Cavazos, Andrés M. Tibabuzo Perdomo, Andrew L. Garfoot, Roberta M. O’Connor, Laura J. Knoll

**Affiliations:** a Morgridge Institute for Research, Madison, Wisconsin, USA; b Department of Medical Microbiology and Immunology, University of Wisconsin—Madison, Madison, Wisconsin, USA; c Microbiology Doctoral Training Program, University of Wisconsin—Madison, Madison, Wisconsin, USA; d Department of Veterinary and Biomedical Sciences, University of Minnesota, St. Paul, Minnesota, USA; University of California, Davis

**Keywords:** *Cryptosporidium parvum*, *Toxoplasma gondii*, STAg, RNA sequencing, ileum, cecum

## Abstract

Animals with a chronic infection of the parasite Toxoplasma gondii are protected against lethal secondary infection with other pathogens. Our group previously determined that soluble T. gondii antigens (STAg) can mimic this protection and be used as a treatment against several lethal pathogens. Because treatments are limited for the parasite Cryptosporidium parvum, we tested STAg as a C. parvum therapeutic. We determined that STAg treatment reduced C. parvum Iowa II oocyst shedding in gamma interferon knockout (IFN-γ-KO) mice. Murine intestinal sections were then sequenced to define the IFN-γ-independent transcriptomic response to C. parvum infection. Gene Ontology and transcript abundance comparisons showed host immune response and metabolism changes. Transcripts for type I interferon-responsive genes were more abundant in C. parvum-infected mice treated with STAg. Comparisons between phosphate-buffered saline (PBS) and STAg treatments showed no significant differences in C. parvum gene expression. C. parvum transcript abundance was highest in the ileum and mucin-like glycoproteins and the GDP-fucose transporter were among the most abundant. These results will assist the field in determining both host- and parasite-directed future therapeutic targets.

## INTRODUCTION

*Cryptosporidium* is an enteric, protozoan parasite of global distribution that causes the diarrheal disease cryptosporidiosis. Cryptosporidium parvum, the most studied species of the 31 species of *Cryptosporidium* reported so far, can cause severe diarrhea in many species, including calves and humans (1). In human immunodeficiency virus (HIV)-infected patients and other immunocompromised individuals, *Cryptosporidium* causes a chronic, debilitating, and sometimes lethal diarrheal disease (2). Additionally, there is an association between *Cryptosporidium* and colorectal cancer (3–6), and recent studies have reported induction of digestive carcinoma in *Cryptosporidium*-infected mice (7–9). Because there is neither a vaccine (10, 11) nor effective therapeutics (12–14) to prevent and treat cryptosporidiosis in immunocompromised patients, characterization of protective immune responses and identification of new therapeutics are medical and veterinary imperatives.

C. parvum is transmitted via the oral-fecal route by consuming food or water contaminated with oocysts, the environmentally resistant stage of the parasite (15, 16). Once inside the host, the oocyst releases sporozoites which invade epithelial cells of the gastrointestinal tract. The parasite undergoes several rounds of asexual replication before transitioning to sexual replication, all within gut enterocytes. Although the parasite is intracellular, it remains extracytoplasmic, forming a feeder organelle by which it extracts nutrients from the host cell. Because of its very reduced metabolism, *Cryptosporidium* relies on the host cell for nucleotides (17, 18), fatty acids (19, 20), and glutaminolysis (21). The life cycle culminates in the production of oocysts that are released into the environment in the feces (17, 22).

During C. parvum infection, the cytokine gamma interferon (IFN-γ) plays a central role in controlling infection in both innate and cell-mediated immune response (23, 24) through a variety of mechanisms. IFN-γ inhibits parasite invasion, changes the intracellular iron (Fe^2+^) concentration in enterocytes that the parasite requires (25), and participates in the clearance of the parasite (24). Patients with IFN-γ deficiency are more likely to develop chronic C. parvum infection (26). While wild-type mice are resistant to C. parvum infection, IFN-γ-deficient mice are highly susceptible (23, 27, 28). Treatment of intact mice with IFN-γ-neutralizing antibodies renders these mice susceptible to C. parvum infection (29). IFN-γ also induces upregulation of the host enzyme indoleamine 2,3-deoxygenase (IDO), inducing tryptophan starvation (30) and killing the parasite. IFN-γ is produced by natural killer (NK) cells (31), macrophages (32), and dendritic cells (33) in response to *Cryptosporidium* infection, and all these cell types have a protective role in the IFN-γ-dependent innate immune response (34). Also, IFN-γ plays a role in *Cryptosporidium* cell-mediated adaptive immune responses, inducing differentiation of naive CD4 T cells to Th1 cells, which secrete more IFN-γ, produce IgG2, and promote cytotoxic-T-cell differentiation (24, 29, 35–37).

To identify novel C. parvum immune responses, we tested a noninfectious extract of soluble Toxoplasma gondii antigens (STAg) for efficacy against C. parvum infection. STAg was previously found to protect against viral, bacterial, and parasitic infections by induction of various innate immune responses. In mice infected with the avian influenza virus H5N1, STAg treatment reduced viral titers in the lungs and induced a strong IFN-γ response resulting in increased survival (38). STAg treatment was also able to prevent experimental cerebral malaria in mice challenged with Plasmodium berghei via induction of interleukin 12 (IL-12) and IFN-γ and subsequent reduction of parasitemia (39). Additionally, STAg treatment provided resistance against Listeria monocytogenes bacterial infection by reducing the bacterial burden in the spleen and liver by stimulation of TLR11 and promoting recruitment of Ly6C CCR2^+^ inflammatory monocytes (40).

Because STAg exhibited immunomodulatory activity protective against a variety of pathogens, for these studies, we tested STAg for anticryptosporidial activity. To mimic immunocompromised patients, we used IFN-γ knockout (IFN-γ-KO) mice to define the IFN-γ-independent host response to C. parvum. Even in the absence of IFN-γ, STAg treatment reduced C. parvum oocyst shedding, indicating that IFN-γ-independent immune responses were effective against the parasite. We were surprised by this IFN-γ independence of STAg, because it has been accepted that STAg elicits host defense via IL-12-mediated induction of IFN-γ, a model that was consistent with our previous coinfection studies (38–40). To understand these novel IFN-γ independence effects of STAg, we performed high-throughput RNA sequencing (RNA-seq) on STAg-treated and C. parvum-infected intestinal tissue to identify components of the immune response responsible for reducing C. parvum oocyst shedding in an IFN-γ-independent manner.

## RESULTS

### STAg treatment reduces oocyst shedding in C. parvum-infected IFN-γ-KO mice.

**FIG 1:**
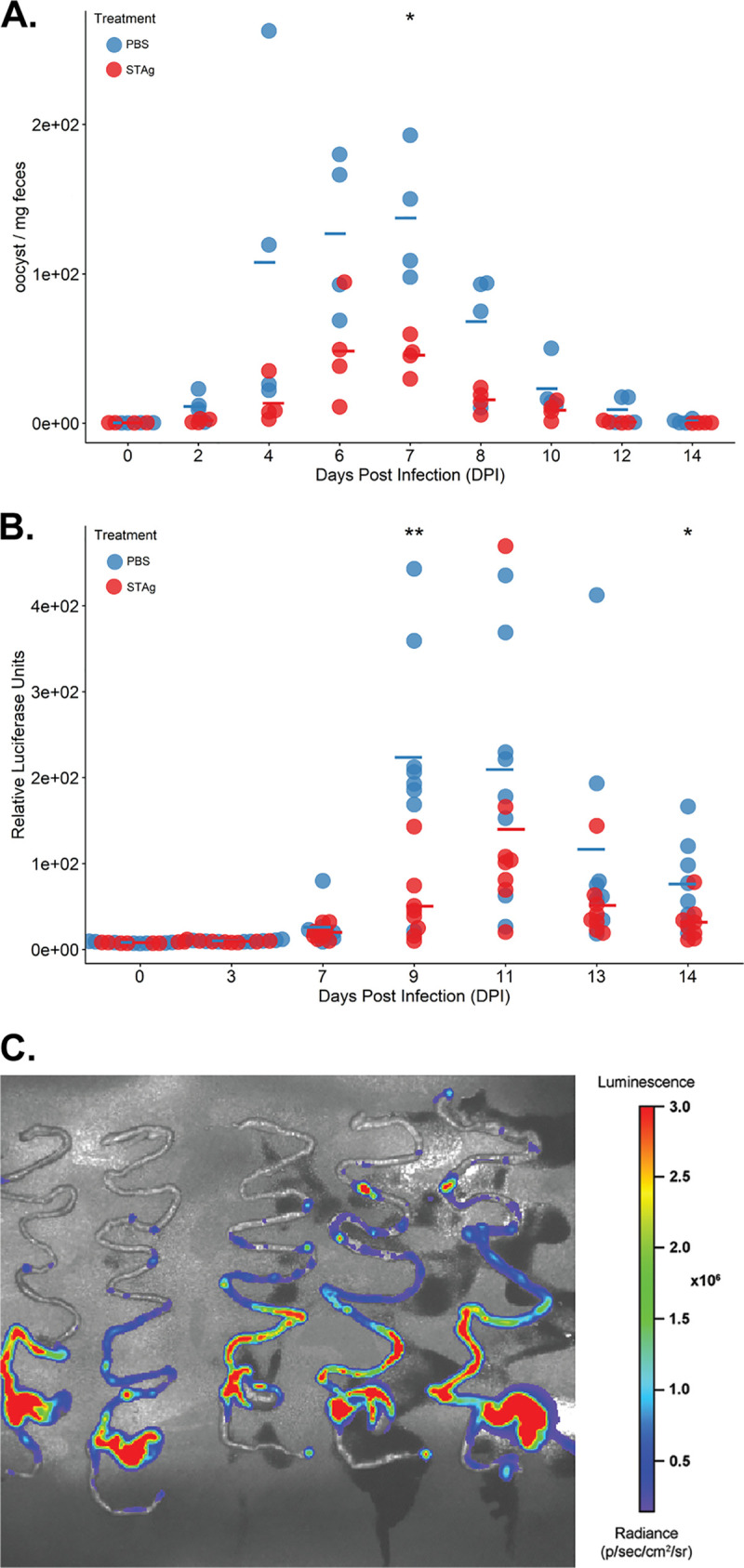
Infection time course of C. parvum infected mice treated with STAg or PBS. (A) Oocyst shedding per dpi in experiment 1 over 14 dpi. *, *P* = 0.020. Oocyst shedding was calculated by qPCR, as described in Materials and Methods. (B) Oocyst shedding per dpi in experiment 2 over 14 dpi. **, *P* = 0.0058; *, *P* = 0.0457. Oocyst shedding was calculated by expression of Nluc, as described in Materials and Methods. Each circle represents a mouse. Open circles, uninfected animals; filled circles, infected animals. Statistic differences were determined using R with analysis of variance (ANOVA). The mean for each group is represented by a line. (C) Bioluminescent imaging of C. parvum in IFN-γ-KO mice throughout the intestinal tract: the intestinal tracts of mice infected for 9 days with Nluc-expressing C. parvum Iowa II strain at 1 × 10^5^ showed a higher signal in the ileum and cecum than the duodenum, jejunum, and colon. The radiance scale is shown.

To evaluate if STAg treatment would affect C. parvum infection, IFN-γ-KO female mice were infected with 1 × 10^7^ C. parvum oocysts of the Iowa II strain by oral gavage and then treated intraperitoneally with 1 mg STAg or PBS control on 1, 3, and 5 days postinfection (dpi). A seminal study previously showed that a lysate of uninfected host cells like human foreskin fibroblasts (HFFs) is indistinguishable from the PBS control when testing induction of IFN-γ by T. gondii tachyzoites (41). Our previous work also showed that an HFF lysate treatment is analogous to the PBS control when testing the protective effect of STAg treatment against influenzas virus infection. Mice that were HFF treated had percent survival, viral titers, and tissue damage similar to those of mice treated with PBS alone (38). Because HFFs were not seen to have a protective effect, we used PBS as the control for the RNA sequencing experiment. Fecal samples were collected every other day from day 0 to 14. Oocyst shedding over time was quantified by quantitative PCR (qPCR) (42). We observed that STAg treatment significantly reduced oocyst shedding compared with the PBS-treated group, indicating that STAg has a therapeutic or immunomodulatory effect against C. parvum infection (experiment 1; *P* = 0.020) (Fig. 1A). The experiment was repeated with IFN-γ-KO mice infected with 1 × 10^5^ nanoluciferase (Nluc)-expressing C. parvum oocysts of the Iowa II strain, and oocyst shedding over time was quantified by Nluc expression (43). STAg treatment again significantly reduced the oocyst shedding (experiment 2; **, *P* = 0.0058 for 9 dpi; and *, *P* = 0.0457 for 14 dpi) (Fig. 1B), confirming our first results (Fig. 1A). There was a slight difference in the peak of oocyst shedding between the two experiments, with the peak of shedding at day 7 for experiment 1 and day 9 for experiment 2. Perhaps the addition of the luciferase gene results in a delay in oocyst shedding. Regardless, from these two independent experiments, we concluded that STAg treatment reduces C. parvum oocyst shedding in the absence of IFN-γ.

### Murine intestinal sections were sequenced to determine the IFN-γ-independent transcriptomic response to C. parvum infection.

To understand the IFN-γ-independent mechanism by which STAg reduces C. parvum oocyst shedding, we wanted to perform transcriptomic analysis of the host. However, we also wanted to simultaneously obtain C. parvum transcripts, so we visualized infection in the entire intestinal tract before we selected a section to sequence. We infected IFN-γ-KO female mice with Nluc-expressing C. parvum and visualized the parasites throughout the mouse intestinal tract using an *in vivo* imaging system (IVIS). Using this technique, Nluc-expressing C. parvum was localized mainly in the cecum and ileum at 9 dpi (Fig. 1C), so these were the sections we chose to sequence.

**FIG 2:**
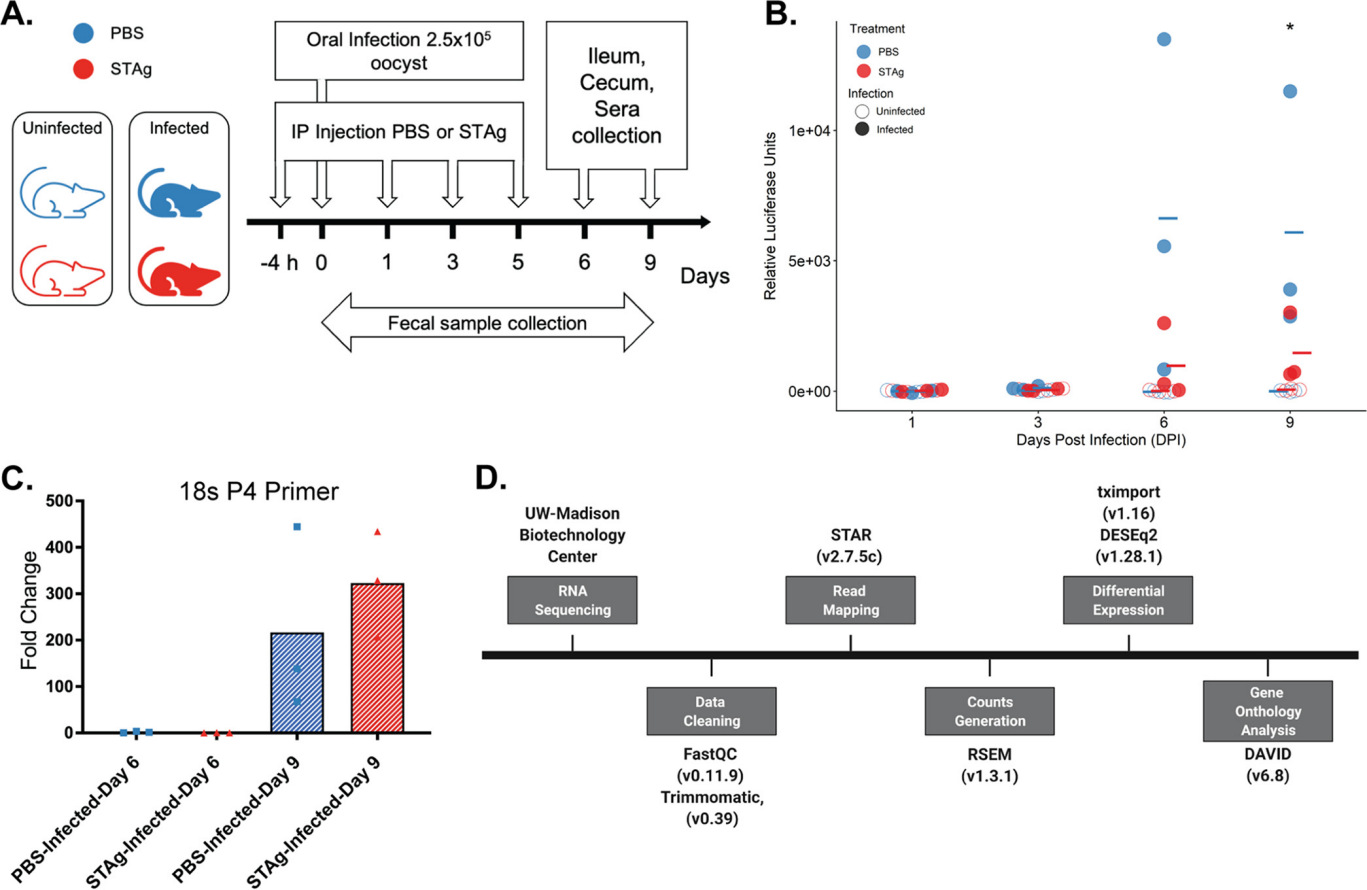
Mouse experiment design and analysis for RNA sequencing. (A) For the RNA-seq analysis, 12 mice (6 infected and 6 noninfected) were treated with PBS or STAg. The treatment was administered 4 h before infection (2.5 × 10^5^ Nluc-expressing C. parvum Iowa II strain oocysts by oral gavage) and 1, 3, and 5 dpi. Fecal samples were collected every other day, and the infection was quantified by Nluc expression. IFN-γ-KO mice were euthanized on 9 dpi, and ileum and cecum samples were collected. (B) Oocyst shedding per dpi in experiment 3 over 9 dpi. Oocyst shedding was calculated by expression of Nluc, as described in Materials and Methods. Significance was determined using R with ANOVA, with *P* = 0.049. The mean for each group is represented with a line. (C) Relative quantification of C. parvum by qPCR using 18S rRNA primers P4 normalized to mouse GAPDH in ileum samples. Values are means from two independent experiments. Fold change was calculated relative to the reference PBS-infected group at day 6. Significance was determined using R with ANOVA. **, *P* = 0.0079 (infected STAg treated, day 6 versus day 9). Each circle represents a mouse. (D) RNA sequencing analysis flowchart listing the programs and packages used for reading and analysis.

To investigate the changes in the transcriptome attributable to STAg during C. parvum infection, mice were infected and treated and samples collected as shown in Fig. 2A. Infected mice treated with PBS shed more oocysts than STAg-treated infected mice (experiment 3; *P* = 0.049) (Fig. 2B), indicating that results in this experiment were analogous to those in experiments whose results are shown in Fig. 1. As C. parvum infects and replicates in enterocytes (15, 44, 45), we collected the epithelial cell layer in the ileum and cecum for transcriptomic analysis. This method allowed us to remove the mucus layer and to concentrate the parasite transcriptome relative to the host transcriptome. We evaluated these samples by qPCR for C. parvum 18S rRNA to determine parasitemia levels. We found that day 9 had significantly more C. parvum 18S rRNA than day 6 (Fig. 2C; also, see Fig. S1 in the supplemental material), so we sequenced day 9 samples. Raw reads were processed and analyzed for differential expression and gene ontology enrichment (Fig. 2D; also, see Table S1).

### Intestinal transcriptomic response to C. parvum Iowa II oral challenge is the most prominent in the host’s ileum.

**FIG 3:**
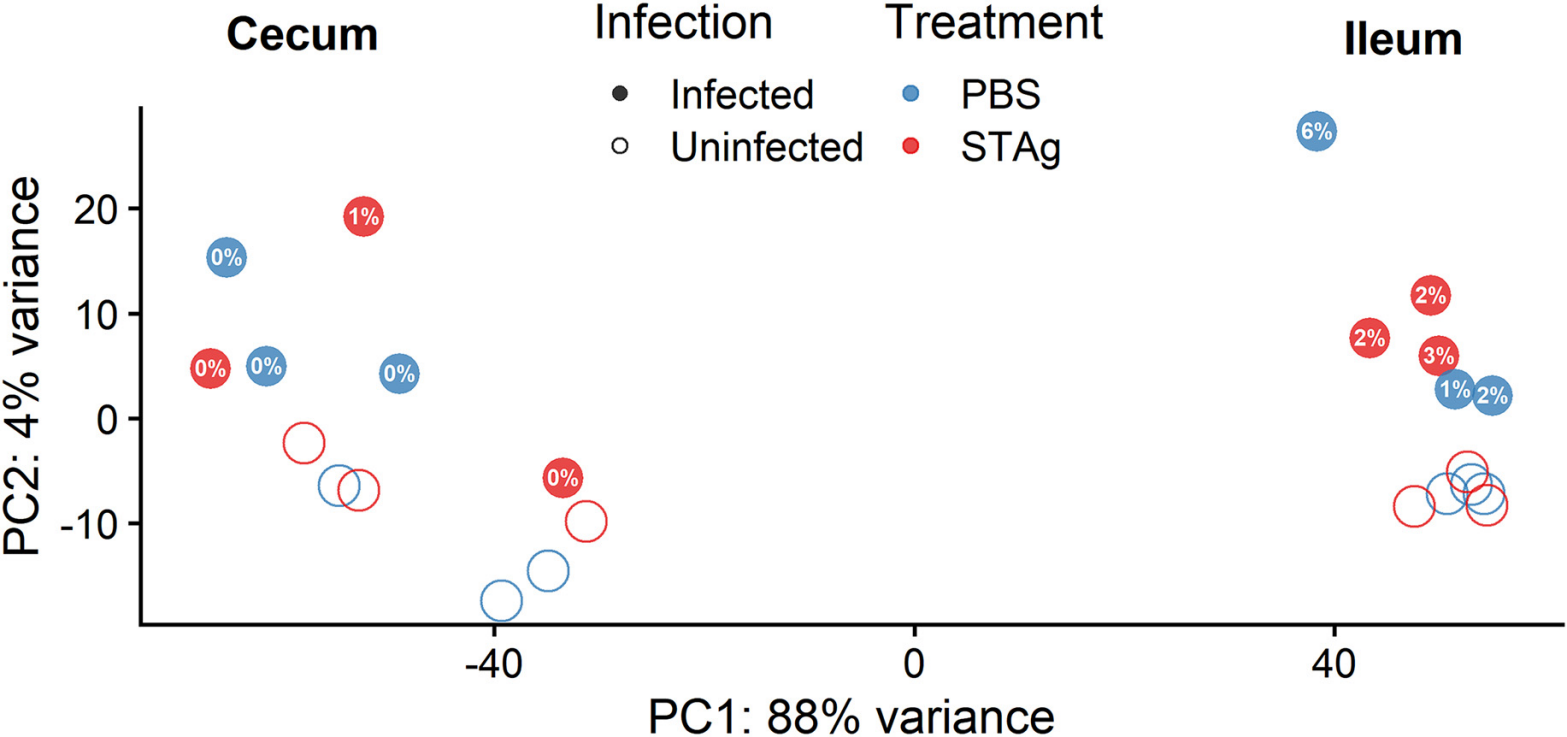
PCA of ileum and cecum samples. PCA plot from normalized values calculated by DESeq2. Mapping of the cecum and ileum to the Mus musculus genome. Percentages in filled circles correspond to percentages of reads uniquely mapped to the C. parvum Iowa II genome up to one significant figure; for more detailed mapped percentages, see Table S1.

A total of 12 differential gene expression comparisons between tissues, treatments, and infection status were conducted for Mus musculus (Table S2) and C. parvum Iowa II (Table S3) transcripts. Principal-component analysis (PCA) was used to visually compare the ileal and cecal transcriptomes of mice treated with STAg or PBS with and without C. parvum infection (Fig. 3). Clustering of normalized values calculated by DESeq2 indicates a clear separation between ileal and cecal samples. Ileal samples from STAg-treated animals (*n* = 6; *n* = 3 per infection group) were clustered by infection status (Fig. 3, right). One of the ileal samples from the PBS-treated infected group does not cluster with the other infected samples, likely due to the percentage of reads mapped to the C. parvum Iowa II genome (5.56% versus 1.21% and 1.66%). The cecal samples did not cluster significantly based on infection or treatment.

**FIG 4:**
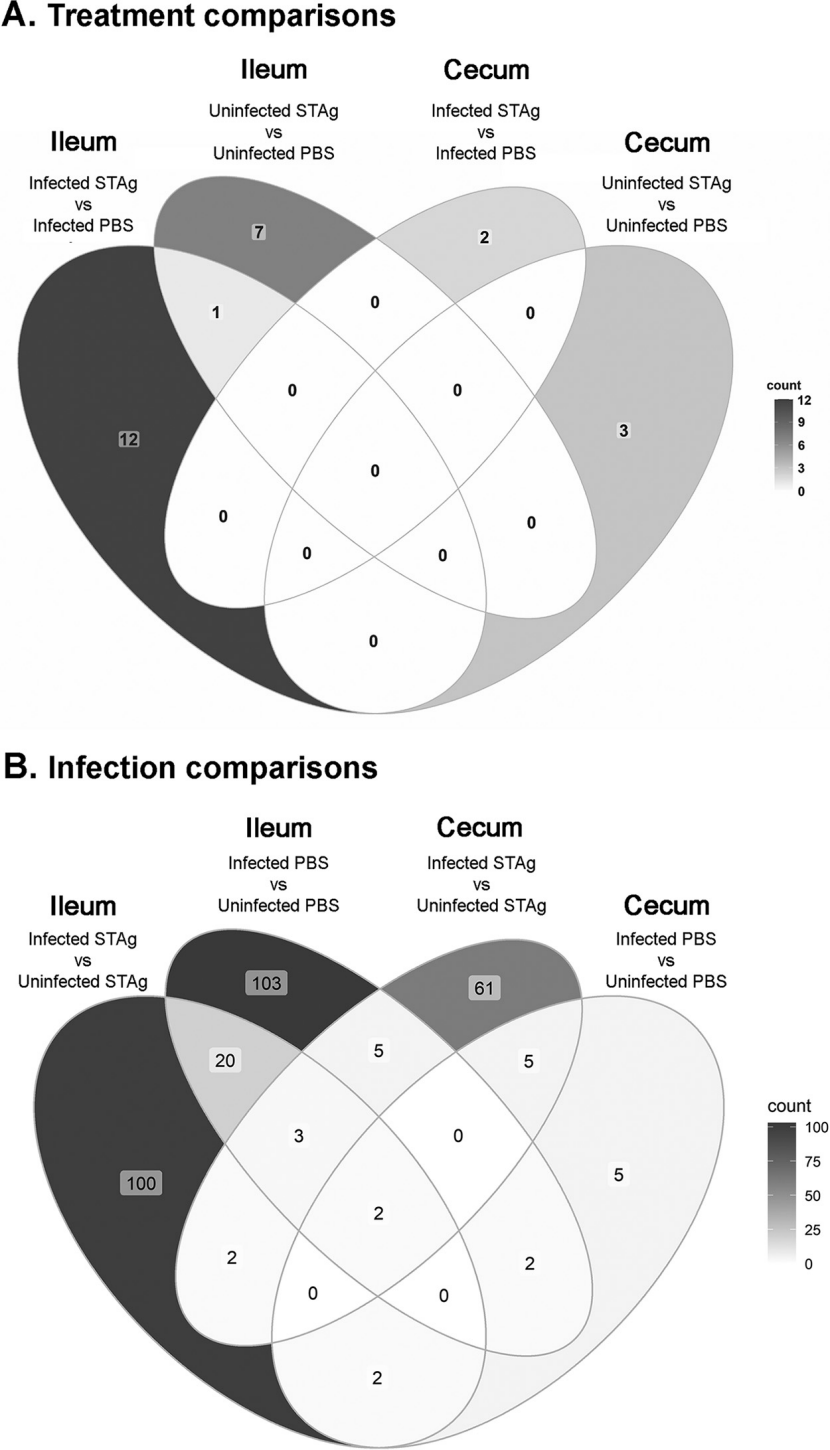
Host differentially expressed genes when comparing treatment (A) and infection (B). The color scale presents higher numbers of DEGs as darker shades of gray.

In terms of the host transcriptomic response to infection and STAg treatment, we observed a low number of differentially expressed genes (DEGs) when comparing the PBS and STAg treatments. In the ilea of infected animals, STAg treatment had 13 significant DEGs compared to PBS treatment. However, in the ceca of infected mice, STAg treatment had only 2 DEGs compared to PBS treatment (Fig. 4A; Table S2, comparisons 1 to 4). Only a single gene, encoding myosin-binding protein C2 (Mybpc2), is shared between comparisons 1 and 2 (Fig. 4A), highlighting that it is less abundant with STAg treatment with or without infection.

In contrast, when we compared the impact of infection, we found the highest number of DEGS. (Fig. 4B; Table S2, comparisons 5 to 8). As the host cell responds to infection, it was expected to have a transcriptomic effect. Whether STAg or PBS treated, more than 100 genes were significantly differentially expressed within the ileum (Fig. 4B) between infected and uninfected animals. Within the STAg-treated mice, a total of 129 genes for the ileum (Fig. S2C) and 78 for the cecum (Fig. S2D) were classified as differentially expressed. Of the 129 genes within the ileum of STAg-treated mice, 100 were exclusive to the ileum (Fig. 4B). Of 78 cecal DEGs between infected and uninfected STAg-treated mice (Fig. 4B), 61 were exclusive to the cecum of STAg-treated animals, and 7 were shared with the ileum of STAg-treated mice. The remaining 10 genes were not exclusive to STAg treatment and were shared with PBS-treated groups: 5 ileum PBS-treated and 5 cecum PBS-treated groups (Fig. 4B). Only 2 genes were shared in every comparison when treatment was constant and infection status was compared: those encoding keratin 13 (Krt13) and cytosolic phospholipase A2 gamma (Pla2g4c). Krt13 and Pla2g4c are more abundant in infected mice regardless of treatment status. In the rest of the comparisons, only a low number of transcripts were shared (Fig. 4A). PBS treatment allowed us to observe the natural IFN-γ-independent host responses. Seven genes are exclusive to these control infections (Fig. S3A).

Overall, the number of DEGs was higher for the ileum (Fig. 4B; Fig. S2A and C) than for the cecum (Fig. 4B; Fig. S2B and D). These results are not due to unmapped reads, as we observed, on average, ∼79% of the reads of both tissues to be uniquely mapped to the host genome. Because the percentage of reads that mapped to the C. parvum Iowa II genome is considerably higher in the ileum than the cecum (Fig. 3; Table S1), it is not surprising that this tissue had the highest number of significant DEGs regardless of treatment.

### Gene Ontology analysis shows differentially expressed immune responses to C. parvum infection based on treatment.

**FIG 5:**
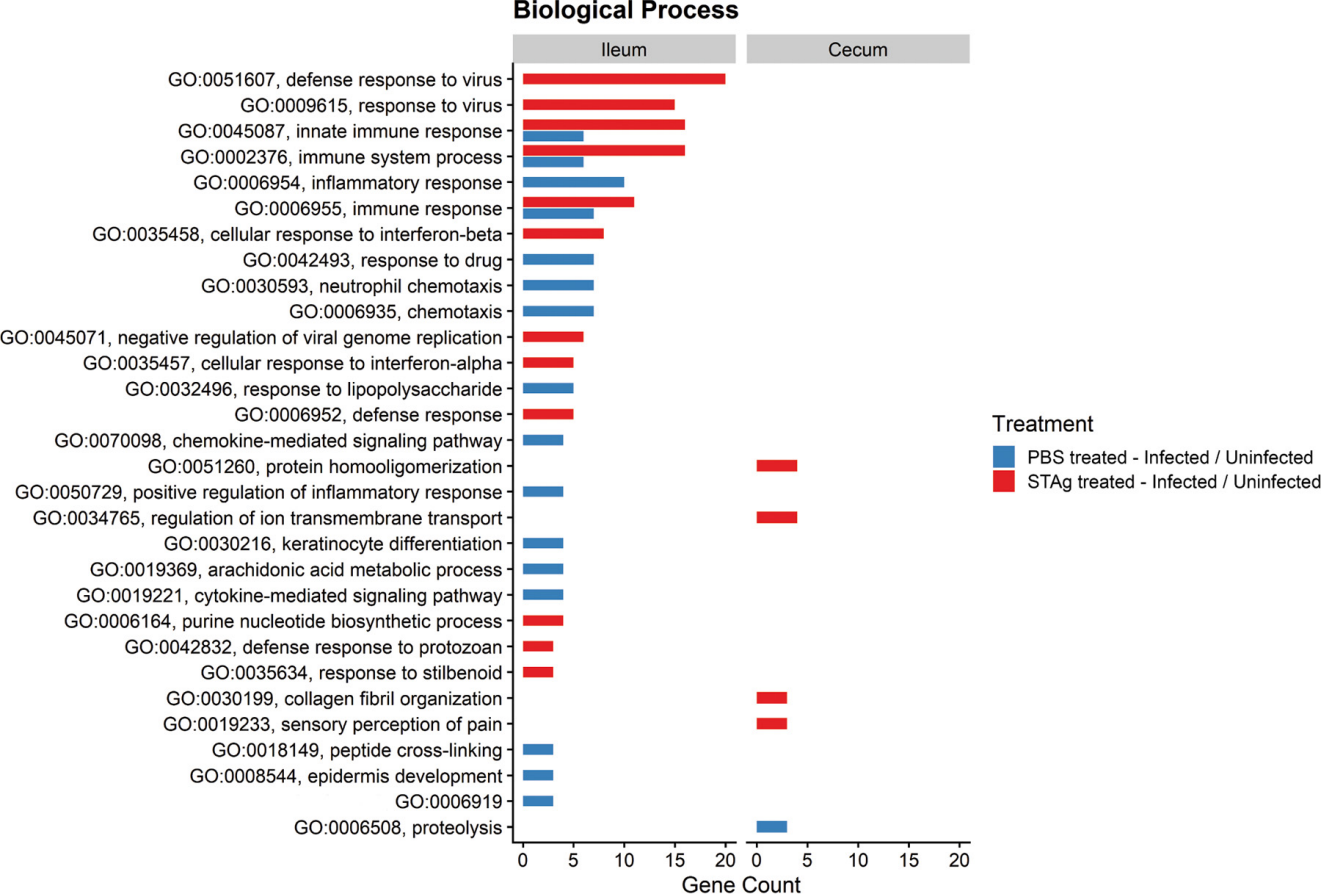
Host gene ontology (GO) of biological process in STAg- and PBS-treated mice. DEGs in comparisons 5 to 8 were analyzed for GO enrichment of biological process, using the Database for Annotation, Visualization, and Integrated Discovery (DAVID, v6.8). Only GO terms populated by 3 or more DEGs were included in these visualizations (*P* < 0.05). GO:0006919 stands for “activation of cysteine-type endopeptidase activity involved in apoptotic process.”

To further explore the transcriptomic response to C. parvum Iowa II infection and STAg treatment, we used Gene Ontology (GO) enrichment analysis through the Database for Annotation, Visualization, and Integrated Discovery (DAVID), v6.8 (46, 47). Genes that were differentially expressed between infected and uninfected mice with PBS or STAg treatment (comparisons 5 to 8 in Table S2) were analyzed for enrichment of three GO term categories: cellular component (Fig. S4), molecular function (Fig. S5), and biological process (Fig. 5). In comparison to the cecum of uninfected STAg-treated mice, the ceca of infected STAg-treated mice were enriched for cellular component GO terms skewed toward the periphery of the host cells—host membrane (GO:0016020), extracellular space (GO:0005615), and extracellular region (GO:0005576)—when infection status was compared (Fig. S4). In the ilea of infected STAg-treated mice, compared to the uninfected STAg-treated mice, DEGs were associated with the mitochondrion (GO:0005739), the extracellular region (GO:0005576), and the endoplasmic reticulum (GO:0005783) (Fig. S4). Cellular component GO terms had the highest level of enrichment for the cecum samples from STAg-treated animals, which was not observed in the biological process or molecular function GO terms (Fig. S5; Fig. 5). Due to the localization of the parasite on the periphery of the host cell, it is encouraging that we observe GO terms that are related to the extracellular space and cellular trafficking when comparing infection. We observed major enrichment of molecular function GO terms related to purine metabolism and RNA binding in the ilea of mice that were infected and STAg treated, which was not observed in the ilea of mice that were PBS treated (Fig. S5).

As expected, the biological process GO terms showed enrichment for host immunity in the ilea of mice treated with STAg (Fig. 5). As we tested our hypothesis in IFN-γ-KO mice, we expected the signaling to be IFN-γ independent. The innate immune response was significantly associated with type I interferon response, including IFN-α signaling (GO:0035457) and response to IFN-β signaling (GO:0035458), and with downstream targets like defense response to virus (GO:0051607) and response to virus (GO:0009615) (Fig. 5).

### Transcript abundance comparisons show immune response and host metabolism changes.

**FIG 6:**
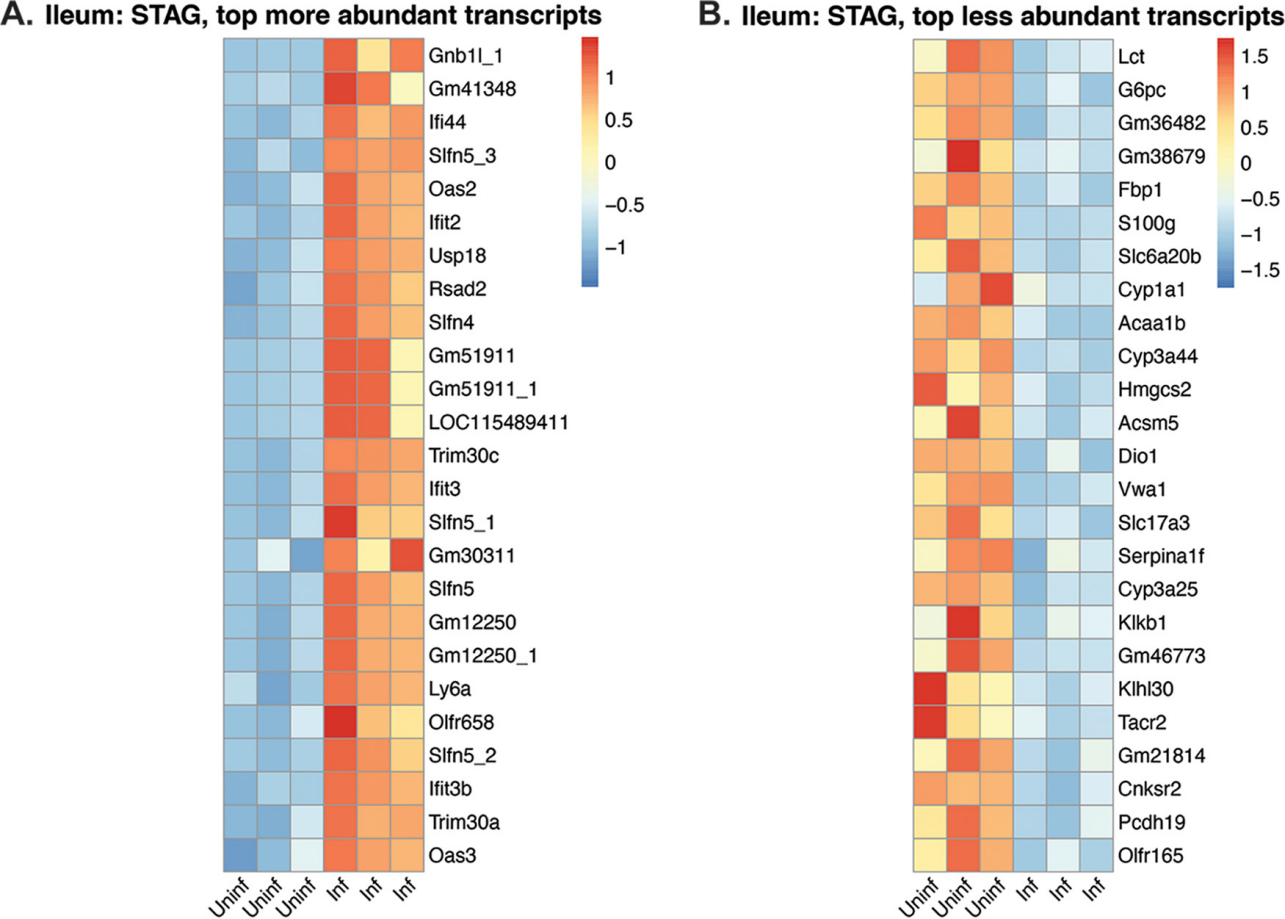
Heat map of differentially expressed genes in the ilea of mice treated with STAg (comparison 5). The top 25 more (A) and less (B) abundant transcripts (ranked by log_2_ fold change). Each column represents a biological replicate.

As the DEGs in the cecum were highly variable among biological replicates, we focused on ileal samples for further analysis. To compare the abundance of these significant genes across the 6 biological replicates per treatment group, we generated heat maps charting the normalized reads per animal (Fig. 6A; Fig. S6). We found 37 genes related to IFN type I in mice infected and treated with STAg (Table 1; Fig. 6A) but not in mice infected and treated with PBS (Fig. S6). These included genes for the following: oligoadenylate synthetase (OAS) (eight genes), DDx60 (48), Dhx58 (also known as Lpg2) (49), Mtx2, Tap1, Trim 30a-b-c, the ubiquitin-specific peptidase 18, seven interferon-induced proteins, immunity-related GTPase family M member 1 and 2, and RING finger proteins 213 and 225 (50, 51). Similarly, 6 members of the Schlafen gene family (SLFN) were present in higher abundance in STAg-treated infected mice versus STAg-treated uninfected mice (comparison 5) (Table 1). To evaluate type I interferon responses earlier in infection, we examined the abundance of interferon-induced protein 44 (IFI44) and OAS-3 at 6 and 9 dpi by qPCR. As expected, IFI44 and OAS-3 were significantly more abundant in infected STAg-treated mice than uninfected mice at 9 dpi, but the expression differences were not significant at 6 dpi (Fig. S7).

**Table 1:** Transcripts associated with IFN type I present in the ileum of STAg-treated mice (infected vs uninfected)

Gene ID	Log_2_ fold change	Gene name
Ddx60	2.45	DEAD (Asp-Glu-Ala-Asp) box polypeptide 60
Dhx58	2.82	DEXH (Asp-Glu-X-His) box polypeptide 58
Ifi204	3.09	Interferon-activated gene 204
Ifi44	4.95	Interferon-induced protein 44
Ifit1	2.55	Interferon-induced protein with tetratricopeptide repeats 1
Ifit1bl1	2.53	Interferon-induced protein with tetratricopeptide repeats 1B like 1
Ifit2	4.68	Interferon-induced protein with tetratricopeptide repeats 2
Ifit3	3.76	Interferon-induced protein with tetratricopeptide repeats 3
Ifit3b	3.48	Interferon-induced protein with tetratricopeptide repeats 3B
Igtp	3.22	Interferon-γ-induced GTPase
Irgm1	2.13	Immunity-related GTPase family M member 1
Irgm2	2.18	Immunity-related GTPase family M member 2
Mx2	3.17	MX dynamin-like GTPase 2
Oas1a	2.47	2'-5' oligoadenylate synthetase 1ª
Oas1b	2.88	2'-5' oligoadenylate synthetase 1B
Oas1g	2.54	2'-5' oligoadenylate synthetase 1G
Oas1h	2.74	2'-5' oligoadenylate synthetase 1H
Oas2	4.70	2'-5' oligoadenylate synthetase 2
Oas3	3.39	2'-5' oligoadenylate synthetase 3
Oasl1	2.06	2'-5' oligoadenylate synthetase-like 1
Oasl2	2.75	2'-5' oligoadenylate synthetase-like 2
Rnf213	2.11	Ring finger protein 213
Rnf225	2.33	Ring finger protein 225
Slfn1	3.16	Schlafen 1
Slfn10-ps_3	2.49	Schlafen 10, Pseudogene
Slfn2	2.52	Schlafen 2
Slfn4	4.11	Schlafen 4
Slfn5	3.72	Schlafen 5
Slfn5_1	3.74	Schlafen 5_1
Slfn5_2	3.53	Schlafen 5_2
Slfn5_3	4.82	Schlafen 5_3
Tap1	2.10	Transporter 1, ATP-binding cassette, subfamily B (MDR/TAP)
Trim30a	3.48	Tripartite motif-containing 30A
Trim30b	2.26	Tripartite motif-containing 30B
Trim30c	3.80	Tripartite motif-containing 30C
Usp18	4.41	Ubiquitin-specific peptidase 18

Curiously, 2 transcripts related to gluconeogenesis were identified in lower abundance in the ileum of infected and STAg-treated mice than in their uninfected counterparts (Fig. 6B), glucose-6-phosphatase catalytic subunit (G6pc) and fructose 1,6-bisphosphatase (Fbp1). There have been sporadic reports that juvenile animals (52) or senior human hosts (53) with C. parvum infection seem to have a lower tolerance for lactose, and we are the first to observe lactase (Lct) transcript to be in lower abundance in infected mice (Fig. 6B). In the PBS-treated mice, transcripts for G6pc, Fbp1, and Lct were also less abundant, but they did not achieve the set threshold limits. For Fbp1, the log_2_ fold change was −1.8, and for Lct, the log_2_ fold change was −1.9. While G6pc did make the −2 log change cutoff (−2.7), due to variability, G6pc did not meet the adjusted *P* value (*P*_adj_) threshold (*P* = 0.07). To evaluate these metabolic changes earlier during infection, we examined the abundance of Fbp1 and Lct at 6 and 9 dpi by qPCR. While Fbp1 was significantly lower in infected animals at 9 dpi, the differences were not significant at 6 dpi (Fig. S7). Lct trended lower in infected animals at 9 dpi, but no differences were seen at 6 dpi (Fig. S7). These results suggest that infection with C. parvum may modulate host carbohydrate metabolism away from gluconeogenesis and disaccharides like lactose during the infection peak. It will be interesting to further investigate if C. parvum infection reduces the expression of these enzymes in other hosts or *in vitro* models.

**FIG 7:**
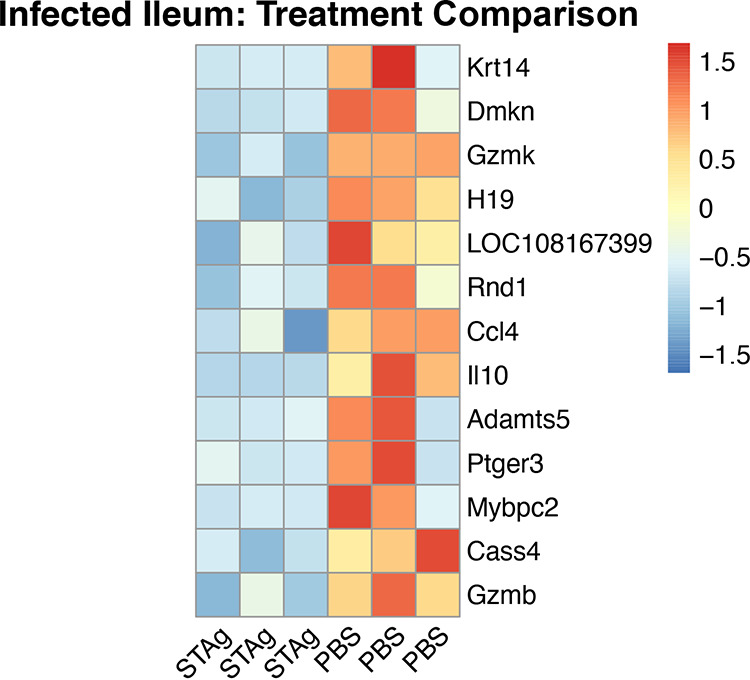
Heat map of differentially expressed genes in the ileum of infected mice treated with STAg or PBS. Thirteen genes (ranked by log_2_ fold change) were found to be differentially expressed in the ileum between infected mice treated with PBS and those with STAg. Each column represents a biological replicate.

While comparing the PBS and STAg treatments within infected animals, we observed 13 DEGs (Fig. 4A). STAg-treated mice have a lower abundance of IL-10, chemokine 4 (Ccl4), prostaglandin E receptor 3 (Ptger3), granzyme B (Gzmb), granzyme K (Gzmk), keratin 14 (Krt14), and others than PBS-treated mice (Fig. 7). These results highlight that STAg treatment reduced the abundance of some inflammatory responses elicited by C. parvum.

### C. parvum transcript analysis shows no significant differences in gene expression between PBS and STAg treatments.

An analysis was conducted to determine if STAg treatment had any effect on the transcriptome of C. parvum (Table S3). When a PCA exploration for reads that were aligned to the C. parvum Iowa II genome was conducted, we observed clustering of the ileum but not the cecum samples (Fig. S8). The percentage of reads that mapped uniquely to the C. parvum Iowa II genome was significantly lower than host mapping, a result that we were expecting on the basis of other similar studies (54). On average per sample, the ileum had more transcripts that mapped to the C. parvum Iowa II genome than the cecum (Table S1). Differential expression analysis of the parasite transcriptome was performed in the same manner as for the host transcriptome, but there were no significantly differentially expressed genes between PBS- and STAg-treated samples. These results indicate that the ability of STAg to lower C. parvum oocyst shedding was more likely due to changing the host immune response than a direct effect on the parasite.

The most abundant C. parvum Iowa II transcripts within the ileum are listed in Tables 2 and 3. The presence of transcripts encoding *Cryptosporidium* mucin-like glycoproteins (55) such as cpMuc5 (cgd2_430) (56), cpMuc7 (cgd2_450), and cpMuc3 (cgd2_410) were observed in STAg-treated animals (Table 2) and gp40 and the mucin cgd7_4020 in PBS-treated animals (Table 3). For both treatments, the parasite GDP-fucose transporter (CGD3_500) was among the 10 most abundant transcripts.

**Table 2:** Top 10 more abundant *C. parvum* transcripts in STAg-treated ileum^*a*^

				Counts for:		
	CryptoDB_ID	Transcript name	Average counts	Mouse 1	Mouse 2	Mouse 3
1	cgd6_10	Proteophosphoglycan	5938	4943	5784	7088
2	cgd4_3630	Cross-beta structure silk protein 1	3007	2621	3043	3357
3	cgd2_410	Signal peptide-containing protein-Mucin 3	2099	1659	1984	2654
4	cgd6_1070	Uncharacterized protein	1989	1747	1975	2246
5	cgd4_3410	RNA recognition motif/WW Domain-containing protein	1877	1515	1837	2281
6	cgd3_550	Major Facilitator Superfamily	1875	1612	1687	2327
7	cgd3_500	GDP-fucose transporter	1582	1306	1596	1844
8	cgd2_430	Signal peptide-containing Protein-cpMUC5	1567	1424	1591	1687
9	cgd8_550	Uncharacterized protein	1512	1239	1542	1755
10	cgd2_450	Signal peptide-containing protein-cPMUC7 CIPW_000002748	1447	1286	1485	1569

a For a complete list, see the supplemental material.

**Table 3:** Top 10 more abundant *C. parvum* transcripts in PBS-treated ileum^*a*^

				Counts for:		
	CryptoDB_ID	Transcript name	Average counts	Mouse 1	Mouse 2	Mouse 3
1	cgd6_1080	Glycoprotein gp40	50,954	20891	34483	97488
2	cgd2_1373	Unspecified product	23,847	17477	15032	39033
3	cgd6_3990	Elongation factor 1-alpha	22,218	9455	15754	41446
4	cgd8_3520	Uncharacterized secreted protein	21,677	9928	16857	38247
5	cgd5_1470	Nucleoside diphosphate kinase	17,366	7388	12296	32415
6	cgd7_4020	Cryptosporidial mucin	16,472	6621	12245	30549
7	cgd1_3810	Uncharacterized protein	16,058	7149	12082	28944
8	cgd3_510	GDP-fucose transporter	14,106	6146	10245	25928
9	cgd5_3160	Actin	13,058	5118	8647	25408
10	cgd6_5410	Uncharacterized secreted protein	11,013	5104	8578	19357

a For a complete list, see the supplemental material.

## DISCUSSION

While nitazoxanide has some efficacy in immunocompetent adults, it is not effective in severely immunocompromised patients (57–59), highlighting the need to develop interventions effective in the absence of normal immune responses. To mimic the human immunocompromised state, we used IFN-γ-KO mice to define the IFN-γ-independent host response to C. parvum. We found that STAg treatment reduced C. parvum infection in IFN-γ-KO mice (Fig. 1) and performed transcriptomic analysis to determine this novel IFN-γ-independent mechanism of STAg treatment. IFN-γ-KO mice have a higher number of significant DEGs when infections in tissues treated with PBS and STAg are compared (Fig. 4B), suggesting that treatment alone is not sufficient to elicit a host gene expression change. C. parvum infection likely acts synergistically with STAg treatment to elicit the type I interferon immune response. It is interesting that the intestinal parasitemia as measured by qPCR of C. parvum 18S rRNA was not reduced by STAg treatment (Fig. 2C), but the shedding was (Fig. 2B). Perhaps the host response elicited by STAg preferentially targets oocyst shedding and not parasite replication. However, the difference could also be due to the nature of the two assays. While the luciferase assay detects only viable parasites that express the Nluc reporter, the qPCR assay detects viable and dying/dead parasites prior to the degradation of their genomic DNA.

Most of the DEGs in the ileum were classified as belonging to the type I interferon response and its downstream effectors, typically known for their antiviral activity (Table 1; Fig. 6A). IFNs are a multigenic family of cytokines that regulate different aspects of the immune response. There are three classes of IFN, types I, II, and III, which recognize distinct receptors on cell membranes (60). IFN signaling induces transcription of hundreds of IFN-stimulated genes that further the immune response against viral infection. The IFN type I response is essential in the immune response against viruses (61) but its role in *Cryptosporidium* infection has been poorly characterized. In 2009, Barakat et al. were the first to describe the IFN type I response to *Cryptosporidium* infection in human and mice enterocytes *in vitro* (62). Upregulation of long noncoding RNAs influences the IFN type I response to C. parvum in an *in vitro* murine model (51). IFN type I genes were upregulated in lung organoids microinjected with oocysts at 72 h postinjection (63). Our study is the second *in vivo* transcriptome analysis to report more abundant IFN type I transcripts during C. parvum infection.

Among the IFN-stimulated genes, we found in high abundance eight oligoadenylate synthetase (OAS) and six Schlafen genes in infected and STAg-treated mice (Table 1). OASs synthesize short oligomers that activate RNase L to cleave cellular and viral RNAs (64, 65), participate in apoptosis, and control cellular growth (66). There are at least a dozen OAS proteins in mice and four in humans. OAS-like proteins consist of one OAS domain and two ubiquitin-repeat domains (UBL). Humans have one OAS-like protein, while mice have two OAS-like proteins, OASL1 and OASL2. Murine OASL1 has a context-dependent role in the antiviral response; in the early stages of infection, it forms stress granules trapping viral RNAs, but in later stages of infection, it downregulates the IFN response (61, 64, 67). The murine Schlafen family has 9 proteins with 5 human homologs. Schlafen proteins have been described to have many potential roles in cell processes, including but not limited to cell differentiation, inhibition of anchorage-independent growth, and regulation of the immune response (68, 69). SLFN2 has been shown to regulate type I interferon response via the NF-κβ pathway (70). SLFN3 and its human analog SLFN12 are critical in regulating intestinal epithelial differentiation (43, 44). Of this family, the most abundant transcript in our transcriptomic data set was Schlafen 4 (Slfn4) (71). A previous study showed a reduction in the recruitment of inflammatory macrophages in SLFN4-overexpressing mice, suggesting that SLFN4 is a negative regulator of monocytopoiesis (72). It will be interesting to further explore the relationship between SLFN4 and C. parvum infection.

Using GO enrichment analysis, we found an enrichment of immunological terms downstream of IFN-α/β signaling (Fig. 6). Curiously, no change in IFN-α/β transcript abundance was observed. This change in the abundance of the effectors versus the signaling molecules themselves is probably due to the late time point selected. We chose the 9-dpi time point to coincide with the peak C. parvum Iowa II oocyst shedding to maximize the number of C. parvum transcripts in the data sets (Fig. 2C; Fig. S1). Future experiments will analyze the IFN-α/β signaling pathways throughout the time course of infection to determine the level of protection type I signaling confers against C. parvum infection.

STAg-treated mice had a lower abundance of IL-10 (Fig. 7). IL-10 is an anti-inflammatory cytokine; its role is to modulate a balance between pathology and protection against a rampant immune response (73, 74). IL-10 is elevated in the stool of *Cryptosporidium*-infected Haitian children (73) as well as in patients coinfected with C. parvum and HIV (75). In animal models, IL-10 knockout mice are more resistant to C. parvum infection (76), but anti-IL-10 antibody treatment in calves does not reduce C. parvum shedding (77). We did not see significant differences in serum IL-10 levels between treatment groups at either day (Fig. S9). There are likely differences between localized/tissue responses and cytokines in serum. We have seen this phenomenon before with *Eimeria* intestinal infection and the IL-10 response (78).

STAg-treated mice also had a lower abundance of Ccl4, the prostaglandin receptor Ptger3, and granzymes B and K (Fig. 7), which have all previously been implicated in *Cryptosporidium* infection. Ccl4 is a chemokine independent of IFN-γ whose expression level increases during C. parvum infection compared to control mice (33). While the differences in prostaglandin receptors have not previously been seen, high levels of prostaglandins have been observed in C. parvum infection, which leads to increased mucosal activity and downregulation of inflammatory cytokine production (79, 80). Granzymes induced by NK cells or CD8^+^ cytotoxic T cells (81) have been found in high abundance in mice and humans infected with *Cryptosporidium*, inducing cell death and cytolysis of infected epithelial cells. They are also associated with apoptosis and colon lesions in AIDS patients coinfected with *Cryptosporidium* (82–85).

Even though we could not assess C. parvum differential expression, we report the most abundant C. parvum transcripts within the ileum. Among those transcripts, we found the GDP-fucose transporter (CGD3_500) to be abundant in our infected animals, regardless of treatment. In other apicomplexan parasites like Toxoplasma gondii, O-fucosylated glycoproteins can form assemblies that are associated with nuclear pore complexes (86). For both T. gondii and Plasmodium falciparum, O-fucosylation of thrombospondin-like repeats is required for processing proteins relevant to host cell invasion (87, 88).

We also found transcripts for parasite mucins in high abundance. Mucins are glycoproteins that have an amino acid composition consisting of 20% to 55% serine, threonine, and proline residues, with extensive chains of O-linked carbohydrates (55) forming homo-oligomers. Mucins have been reported both in the host and in some gastrointestinal parasites. *Cryptosporidium*’s mucins are important for attachment and invasion to the host cell (55) or evasion of the host immune system (89). Many essential *Cryptosporidium* proteins are mucin-like proteins such as gp40/15 (90), cpClec, gp30, and gp900 (91). There is a genetic locus of seven mucins named cpMuc1-7, which suggests that these seven mucins are regulated and expressed in a coordinated fashion with a role in the same biological processes (55). C*ryptosporidium* mucins have high polymorphism between species and genotypes, which suggests that gene products might be important virulence determinants subject to immune pressure (55). Recently, it has been reported that mucins play a role in tissue tropism between gastric and intestinal *Cryptosporidium* species and also determine differences in host range among *Cryptosporidium* species (92, 93). Further investigation is required for the validation of the differential expression of C. parvum transcriptomic response to STAg treatment.

We did not find significant differences in transcript abundances when comparing STAg and PBS treatments. It is possible that STAg treatment affects only the host transcriptome, while the parasite transcriptome remains unaffected. This lack of differential C. parvum expression could also be due to the limitations of coverage and sequence depth of our study. There was a significantly higher mapping of C. parvum reads to the ileum than the cecum samples. Thus, we focused on the most abundant transcripts within the ileum with both treatments in the hope that the C. parvum community can use these *in vivo* data for comparisons with *in vitro* systems. By discovering C. parvum genes that are more abundant during infection of animals that lack IFN-γ signaling, we help define possible alternatives to control infection.

## MATERIALS AND METHODS

### Ethics approval.

All animal studies were carried out in compliance with the Animal Research: Reporting of *In Vivo* Experiments (ARRIVE) guidelines and approved by the Institutional Animal Care and Use Committee (IACUC) of the University of Wisconsin School of Medicine and Public Health. The University of Wisconsin is accredited by the International Association for Assessment and Accreditation of Laboratory Animal Care. All methods and all experimental protocols were approved by the University of Wisconsin IACUC (protocol M005217) as well as the Office of Biological Safety (protocol B00000086).

### Cryptosporidium parvum.

Nanoluciferase (Nluc)-expressing C. parvum Iowa II oocysts were purchased from the University of Arizona.

### STAg preparation.

The STAg was prepared as described originally (94) except for the following changes. ME49 T. gondii parasites were grown in HFFs under mycoplasma-free standard cell culture conditions in Dulbecco's modified Eagle medium (DMEM) supplemented with 10% fetal bovine serum, 1% penicillin-streptomycin, and 2 mM l-glutamine. When the parasites were beginning to lyse the host cells, monolayers were scraped, passed twice through a 27-gauge needle, and pelleted at 500 × *g*. Parasites were washed in phosphate-buffered saline (PBS) without divalent cations and resuspended to 4 × 10^8^ parasites per ml. After sonication with five 30-s pulses, parasites were centrifuged at 100,000 × *g* for 45 min, and supernatants were collected and stored at −80°C until use.

### Mouse infections. (i) Experiment 1.

Eight 4-week-old female C57BL/6 IFN-γ-KO mice (B6.129S7-Ifngtm1Ts/J; The Jackson Laboratory) were infected by oral gavage with 1 × 10^7^ C. parvum Iowa II oocysts and given STAg (1 mg/dose via intraperitonel [i.p.] injection,) or PBS (4 mice per treatment group) at 1, 3, and 5 dpi. Fecal samples were collected every other day from day 0 to 14, and oocyst shedding was quantified by qPCR following the protocol previously published (42).

### (ii) Experiment 2.

Sixteen 4-week-old C57BL/6 IFN-γ-KO mice (B6.129S7-Ifngtm1Ts/J; 8 female and 8 male; The Jackson Laboratory) were infected by oral gavage with 1 × 10^5^ Nluc-expressing C. parvum Iowa II oocysts. Four males and 4 females received STAg, and the other 4 males and 4 females received PBS (1 mg/dose via i.p. injection) at 1, 3, and 5 dpi. Fecal samples were collected every other day from day 0 to 14, and oocyst shedding was quantified by nanoluciferase expression following the protocol previously published (43).

### (iii) Experiment 3.

Twelve 4-week-old female C57BL/6 IFN-γ-KO mice (B6.129S7-Ifngtm1Ts/J; The Jackson Laboratory) were placed into 4 groups: uninfected PBS treated, uninfected STAg treated, infected PBS treated, and infected STAg treated. All mice received pretreatment with STAg or PBS 4 h before infection. Then, 2.5 × 10^5^ Nluc-expressing Iowa II strain oocysts/mice were given by oral gavage to the infected groups. At 24, 48, and 72 h postinfection, the mice were treated with either STAg or PBS. From 6 to 9 dpi, the mice were weighed, and individual fecal samples were collected to evaluate infection levels. On 9 dpi, mice were euthanized. The intestine was isolated and rinsed using a gavage needle and scraped using a single-use sterile cell scraper with sterile 1× PBS. A section of 2 cm of ileum from each mouse was processed. Similarly, the cecum was isolated and rinsed to remove its contents. The cecum bag was opened and scraped. Scraping was performed to isolate the epithelial layer. After microscopic verification, the material was resuspended in 1 ml of 1× Hanks balanced salt solution (HBSS) (Corning). After centrifugation, the pellet was resuspended in 1 ml of TRIzol (Ambion), vortexed, and stored at −80°C.

### (iv) Generation of RNA and RNA sequencing.

We used the University of Wisconsin—Madison Biotechnology Center's Gene Expression Center Core Facility (research resource identifier [RRID]: SCR_017757) for RNA isolation and RNA library preparation and the DNA Sequencing Facility (RRID: SCR_017759) for sequencing and demultiplexing of reads. Libraries were prepared for poly(A) enrichment (Illumina TruSeq stranded mRNA). Samples were run in duplicate in two lanes of a 2 × 100-bp S1 Flowcell (Novaseq6000). On average, a total of 41.5 million reads (minimum, 36 million; maximum, 49.6 million) paired-end 150-bp reads per sample were generated. The quality of the reads was determined (FastQC v0.11.9) (95), a threshold of 34 was selected, and only reads that met the threshold were used for further analysis.

### (v) Transcriptome assembly and differential expression analysis.

We trimmed the data to remove low-quality reads (Trimmomatic, v0.39) (96) Mapping reads to two genomes, Mus musculus strain C57BL/6 (GRCm38.p6, release38; NCBI) and C. parvum (Iowa II, release 49; CryptoDB) was conducted (Spliced Transcripts Alignment to a Reference program, v2.7.5c) (97, 98). Default parameters were selected with the following exceptions: maximum mismatch (2 bp), minimum intron length (20 bp), and maximum intron length (100,000 bp). Quantification of mapped reads and the generation of a counts table were conducted (RSEM v1.3.1) (99). Counts were imported into R (tximport v1.16) (100), and differential expression analysis was conducted (DESEq2 v1.28.1) (101). The log-transformed DESeq2 values were used for the generation of PCA plots. For reads mapped to the host genome, we selected the following thresholds: false discovery rate, 10%; adjusted *P* value, <0.05; and absolute log_2_ fold change, >2. If a differential expression gene met those parameters, we called that gene significantly differentially expressed (Data Sets S1 to S12). Given that the average C. parvum Iowa II percentage mapping (ileum, 2.58%; cecum, 0.33%) was significantly lower than the host mapping (ileum, 78.92%; cecum, 77.95%), we selected only two thresholds for C. parvum Iowa II significance: a false discovery rate of 10% and an adjusted *P* value of <0.05. Lists of significant genes were used as input for gene ontology enrichment analysis using the Database for Annotation, Visualization, and Integrated Discovery (DAVID, v6.8) (46, 47) for visualization of this enrichment; only terms that were populated by 3 or more genes were charted (*P* < 0.05).

### (vi) Luciferase assay.

Mouse infections were confirmed by luciferase measurement of mouse fecal samples following a previously reported protocol (43). Briefly, 20 mg of fecal sample was weighed into a 1.5-ml microcentrifuge tube and homogenized in 1 ml of lysis buffer (50 mM Tris-HCl, 10% glycerol, 1% Triton-X, 2 mM dithiothreitol [DTT], 2 mM EDTA) using 10 to 15 glass beads (3 mm) and a vortex mixer for 1 min, followed by clarification of lysate by brief centrifugation. One hundred microliters of lysate was mixed with an equal volume of NanoGlo luciferase buffer, prepared with a 1:50 dilution of the substrate (Promega). Three technical replicates per sample were conducted. Luminescence was measured in a Synergy HT Luminator (BioTek).

### (vii) Oocyst purification from mouse feces.

Oocysts were purified from mouse fecal samples using sucrose suspension followed by cesium chloride (CsCl) centrifugation as previously published (43). Mouse feces were suspended in water, homogenized, and passed through two filters. This filtered suspension was mixed 1:1 with aqueous sucrose solution (specific gravity, 1.33) and centrifuged at 1,000 × *g* for 5 min. Oocysts were collected from the supernatant and suspended in 0.85% NaCl saline solution. Then, 0.5 ml of this preparation was overlaid onto 0.8 ml of 1.15-specific-gravity CsCl and centrifuged for 3 min at 16,000 × *g*. Oocysts were collected from the top milliliter of the sample, washed in 0.85% saline, and resuspended in 1× penicillin-streptomycin antibiotic solution.

### (viii) Quantification of oocysts by immunofluorescence.

Samples were diluted in PBS, fixed with methanol on the well of a glass slide, and incubated with Crypt-a-Glo antibody (Waterborne, Inc.) for 30 min at 37°C. The slide was then gently washed with 1× PBS and allowed to dry. A drop of antifade mounting medium with DAPI (4′,6-diamidino-2-phenylindole) counterstain (Vectashield) and a coverslip were added. The total number of oocyst/well was quantified at 40× by microscopy with a Zeiss Axioplan III equipped with a triple-pass (DAPI-fluorescein isothiocyanate [FITC]-Texas Red) emission cube, differential interference contrast optics, and a monochromatic Axiocam camera operated by Zen software (Zeiss). The number of oocysts per microliter was calculated as an average of at least three microscopic readings.

### (ix) Bioluminescent imaging of C. parvum Iowa II in mice small intestine with an IVIS.

Female IFN-γ-KO mice (*n* = 5) were infected with 1.5 × 10^5^ Nluc-expressing C. parvum Iowa II oocysts via oral gavage. Mice were euthanized 9 dpi, and their small intestines were removed, cleaned, and temporarily stored in PBS to avoid drying of the sample. Before imaging, the PBS was removed and each sample was transferred to a clean petri dish containing the luciferase activity buffer (NanoGlo^®;^ Promega). The sample was incubated in the buffer for 2 min and transferred to an IVIS to collect bioluminescence data for an exposure time of 5 min.

### (x) Cytokine profile.

The concentrations of the cytokines IL-6, IL-10, IL-12, tumor necrosis factor (TNF), and MCP-1 were measured using a mouse inflammation cytokine bead array kit (552364; BD Biosciences). Blood serum was collected for cytokine analysis on 6 and 9 dpi, following the manufacturer’s instructions. Samples were analyzed by flow cytometry with a Thermo Fisher Attune flow cytometer at the University of Wisconsin—Madison Flow Cytometry Core, and concentrations were calculated using FlowJo.

### (xi) Real-time PCR on ileum cDNA targeting host and parasite genes.

Experiment 3 animals were euthanized 6 and 9 dpi. The scraping technique and isolated RNA from the RNA-seq experiment were used to generate cDNA (Invitrogen SuperScript III first-strand synthesis kit) with oligo(dT) primers to enrich for eukaryotic DNA. Real-time qPCR was performed (Bio-Rad iTaq universal SYBR green supermix) on an Applied Biosystems QuantStudio 7 Flex real-time PCR system. Quantification of C. parvum used the following C. parvum 18S RNA primers: P4-F (TAGAGATTGGAGGTTGTTCCT) and P4-R (CTCCACCAACTAAGAACGGCC), published previously (102), and P5-F (CATGGATAACCGTGGTAAT) and P5-R (TACCCTACCGTCTAAAGCTG), published previously (103). Targeting of host genes used the following primers: m-Ifi44-F3 (AGGCGAGTGTGCAGAAGTTT), m-Ifi44-R3 (CTGGAAGCCCTCTTTCAGGT), m-Oas3-F (CAGAGACCAAGAGTGATAAGG), m-Oas3-R (AGGAAGATGACGAGTTCG), m-lctF2 (TGCACCGCTGGAGGTAATTT), m-lctR2 (GAGAACCACGAGGCAGTTCA), m-Fbp1F1 (GCATCGCACAGCTCTATGGT), and m-Fbp1R1 (ACAGGTAGCGTAGGACGACT). Mouse GAPDH was used to normalize gene expression with the primers mGAPDH_F (CTTTGTCAAGCTCATTTCCTGG) and mGAPDH_R (TCTTGCTCAGTGTCCTTGC). Primers were designed using the NCBI primer designing tool and snapGene version 5.3.1. Expression was calculated by normalizing the cycle threshold values (*C_T_*) to that of GAPDH for each sample (Δ*C_T_*). The Δ*C_T_* value was then used to calculate either 1/*C_T_* or the fold change using the ΔΔ*C_T_* method (relative to that of the uninfected PBS-treated mouse group for each sample).

### Data availability.

All raw RNA sequencing data have been deposited in the NCBI Sequence Read Archive (https://www.ncbi.nlm.nih.gov/bioproject/PRJNA726295) under submission ID SUB9557041 and BioProject ID PRJNA726295. RNA sequencing data have been supplied for public availability to HostDB.org and CryptoDB.org. Differential expression analysis output and counts of mapped reads per individual biological replicate are available in Data Sets S1 to S12.
